# Personalized medicine: myth or reality? The position of Russian clinical pharmacologists

**DOI:** 10.1186/1878-5085-4-13

**Published:** 2013-05-11

**Authors:** Anna S Zhestovskaja, Vladimir G Kukes, Dmitry A Sychev

**Affiliations:** 1 Department of Clinical Pharmacology and Propaedeutics of Internal Diseases, I.M. Sechenov First Moscow State Medical University, Trubetskaja 8, Moscow 119991, Russia; 2 Federal State Budgetary Institution, Scientific Centre for Expert Evaluation of Medical Products, Petrovskiy Boulevard 8, Moscow 127051, Russia

**Keywords:** Personalized medicine, Biotransformation, Isoenzymes of the cytochrome P-450

## Abstract

A personalized medicine, a recent trend of clinical pharmacology, makes possible the individual approach to the choice of the drugs and their dosage. According to the results of a study of the activity of different biomarkers, particularly the isoenzymes of the cytochrome P-450, they provide the efficiency and safety of the pharmacotherapy. The activity of the isoenzymes of the cytochrome P-450 determines an individual pharmacological response and depends on many factors, including genetic ones. The biomarkers of the activity of the isoenzymes of the cytochrome P-450 should be tested in the clinical practice settings using the simple and cheap methods, one of the most available is an immunofluorescent assay. The skilled staff and the centers of personalized medicine are necessary for this approach.

## Review

### Personalized pharmacotherapy: traditional approach or new perspectives

There is a long-standing opinion that pharmacotherapy should be personalized, but physicians did not have the method of choice of drugs and their dosage. The necessity of the individual approach to the choice of the drugs is determined by the efficiency of pharmacotherapy (not more than 60% (WHO)) and the increased frequency of the adverse drug reactions (up to fatal cases). In some clinical trials, while drug administration is in standard dosage, some patients had a high serum concentration and the adverse drug reactions developed; other ones had a low serum drug concentration, and as a result, the treatment was ineffective [1].

In consideration of the foregoing, we will try to answer three questions:

– Why the efficiency of pharmacotherapy is not more than 60% in spite of the existence of concepts of evidence-based medicine?

– Why the frequency of the adverse drug reactions is increasing?

– Why when standard dosage of plenty of administered drugs, the range of drug serum concentration is so wide?

**Figure 1:**
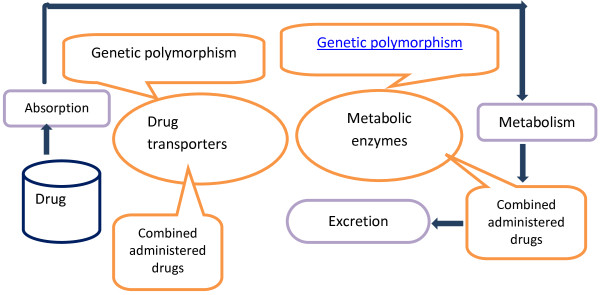
The cycle of ‘movement’ of drugs in the human's organism.

The answers on these questions are in the cycle of ‘movement’ of drugs in the human's organism presented in Figure 1. The key points of this cycle are as follows: an enzyme activity of biotransformation system (mainly the activity of the isoenzymes of the cytochrome P-450) and drug transporters that determine personal pharmacokinetics [2].

Nowadays, it is well known that [3]:

– 40% of drugs are metabolized with the involvement of the isoenzymes of the cytochrome P-450 (CYP450)

– 8% of drugs are prodrugs, and their active ‘drugs’ are active metabolites.

The examples of prodrugs and enzymes of biotransformation participate in the active formation of metabolites.

$$\mathrm{Clopidogrel}\left(\mathrm{Plavix}\right)\to \mathrm{CYP}2C9\to 2-\mathrm{oxoclopidogrel}$$

$$\mathrm{Enalapril}\left(\mathrm{Renitec}\right)\to \mathrm{Carboxyesterase}\to \mathrm{Enalaprilat}$$

$$\mathrm{Azathioprine}\left(\mathrm{Imuran}\right)\to \mathrm{Xanthineoxidase}\to \mathrm{Mercaptopurine}$$

$$\mathrm{Tamoxifen}\left(\mathrm{Tamofen}\right)\to \mathrm{CYP}2D6\to \mathrm{Endoxifen}$$

$$\mathrm{Losartan}\left(\mathrm{Cozaar}\right)\to \mathrm{CYP}2C9\to E-3174$$

$$\mathrm{Spironolactone}\left(\mathrm{Verospiron}\right)\to \mathrm{CYP}3A4\to \mathrm{Canrenone}$$

### Role of genetic polymorphism and isoenzymes of the cytochrome P-450in drug metabolism

**Figure 2:**
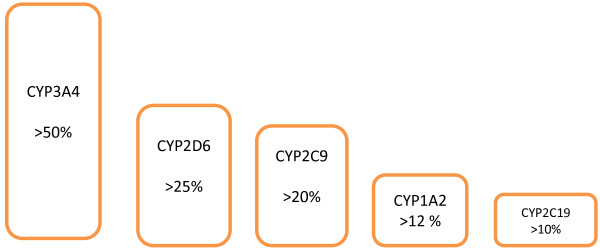
Role of different isoforms of the isoenzymes of the cytochrome P-450 in the drug metabolism.

Ninety percent of drugs are metabolized with the involvement of five isoforms of the isoenzymes of the cytochrome P-450 (CYP3A4, CYP2D6, CYP2C9, CYP1A2, CYP2C19) (Figure 2).

The main factors that influence on the activity of the isoenzymes of the cytochrome P-450 are as follows [4]:

– genetic polymorphism

– combined administered drugs (induction/inhibition)

– severity and character of the main and associated conditions

– qualitative composition of food

**Figure 3:**
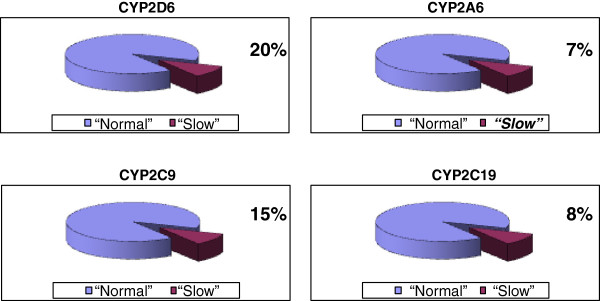
Frequency of ‘slow’ alleles of different isoenzymes of the cytochrome P-450 among the Moscovites.

The genetic polymorphism of the isoenzymes of the cytochrome P-450 determines a personal activity of the isoenzyme and sometimes a risk of the development of the adverse drug reactions. We studied the frequency of ‘slow’ alleles of different isoenzymes of the cytochrome P-450 among the Muscovites (more than 800 genotyping tests were done). Eight to twenty percent of patients, treated with drugs, substrates of isoenzymes of the cytochrome P-450, had analytically determined the risk of development of the adverse drug reactions (Figure 3) [5].

Such results of the genotyping tests let us conclude that when the genetic polymorphism of the enzyme that metabolizes the drug is more than 10% of the cases in the population, the genotyping tests are necessary. But if a drug is the prodrug, from which with the involvement of the isoenzymes of the cytochrome P-450, an active metabolite is formed, the genetic polymorphism may result not in the adverse drug reactions but in the inefficiency of the treatment.

**Figure 4:**
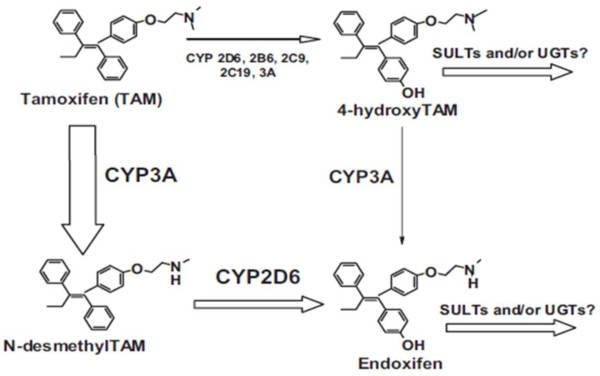
The scheme of metabolite of tamoxifen to the active metabolite, endoxifen.

An example is the anticancer drug, tamoxifen, from which with the involvement of the CYP2D6, its active metabolite, endoxifen, is formed. In women with breast cancer, slow metabolizers of CYP2D6 (carrier state of slow allele CYP2D6*4), endoxifen is formed in the lower levels, and remission period is lower (Figure 4) [6].

Also, a patent example of the genetic polymorphism influence on the development of the adverse drug reactions is the phenomena of the development of Stevens–Johnson syndrome during carbamazepine treatment period in patients with carrier state of HLA-B*1502, and the development of bleedings occurs during warfarin (in standard doses) treatment period in patients with carrier state of allele CYP2C9 [7].

### The isoenzymes of the cytochrome P-450 activity in the range of personalized medicine

So, what should a physician do to make a pharmacotherapy effective and safe? A physician should know how to work in the range of personalized medicine. What is a personalized medicine? According to the Personalized Medicine Consortium (USA), personalized medicine is the use of new methods of molecular analysis for the improvement of prognosis, prophylaxis and treatment of the diseases. The personalized medicine should change the development and realization of measures on prophylaxis and treatment of the diseases. According to experts, the ‘instruments’ of the personalized medicine are genomics, transcriptomics, proteomics and metabolomics [8]. The study of the activity of the isoenzymes of the cytochrome P-450 refers to metabolomics, and in spite of the key role of cytochrome P-450 nowadays, there are no activity tests for it.

**Figure 5:**
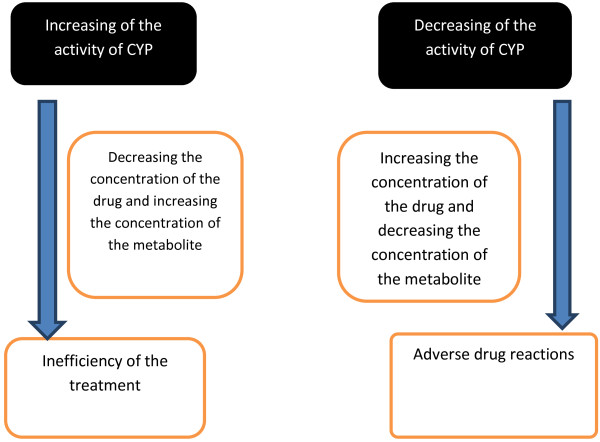
The principle of the activity of the isoenzymes of the cytochrome P-450.

We offer a well-known principle for the isoenzymes of the cytochrome P-450 activity tests: concentration ratio of a substrate and its metabolite. If the concentration of metabolite is more than 50%, the activity of the isoenzymes, involved into the metabolism, is concerned to be high; if it is less than 30%, it is concerned to be low. The scheme of this principle is presented on Figure 5.

Currently, we proved that the activity of the isoenzymes of the cytochrome P-450 is examined more safely not with the help of exogenous but with the endogenous substrates and their metabolites, for example: cortisone (for CYP3A4), cholesterol (for CYP3A4), testosterone (for CYP3A4) and pinoline (for CYP2D6). There are metabolite tests, established on the high-performance liquid chromatography (HPLC) with mass spectroscopy detection. The exogenous substrates for the activity of the isoenzymes of the cytochrome P-450 examination are presented in the Table 1: omeprazole (for CYP2C9), losartan (for CYP2C19), caffeine (for CYP1A2), debrisoquine (for CYP2D6) and midazolam (for CYP3A4). The metabolite tests are established on the HPLC with UV detection.

**Table 1:** The activity of the isoenzymes of the cytochrome P-450 examination

**The isoenzymes of the cytochrome P-450**	**Drugs: the substrates of the isoenzymes**	**The activity examination**
CYP3A4	Slow channel-blocking agents, statins, H1-histamine receptor blockers, cytostatics	• Cortisol/6-beta-hydroxycortisol concentration ratio in the urine [9]
		• 5-Hydroxycholesterol concentration in the blood serum [10]
CYP2D6	Beta-blockers, antidepressants, antipsychotic drugs	• Concentration of the pinoline and its metabolite, hydroxyl-1,2,3,4-tetra-beta-hydrocarboline in the urine [11]
CYP2C9	Oral anticoagulant, NSAID, antidiabetic drugs	• Concentration of losartan and its metabolite, E-3174 in the urine [12]
CYP2C19	Proton-pump inhibitors, clopidogrel, anticonvulsant drugs	• Concentration of 6-hydroxyomeprazole and its metabolite in the blood serum [13]

Thus, we demonstrated that the concentration of the drug in the blood serum depends on the activity of the metabolism enzymes, particularly on the isoenzymes of the cytochrome P-450. If we know the level of the activity of these isoenzymes, we can choose a proper drug and its dosage to achieve a clinical effect and a low percentage of the adverse drug reactions.

## Conclusions

### Centers of personalized medicine: implementation instruments of personalized medicine in clinical practice

**Figure 6:**
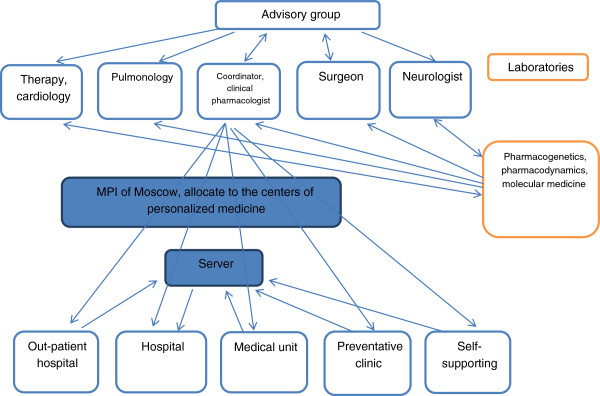
Work layout of the center of personalized medicine.

To promote a methodology of the personalized medicine and evaluation methods of the activity of the isoenzymes of the cytochrome P-450 in clinical practice, federal and regional centers of personalized medicine are necessary. The workflow of the center of the personalized medicine is presented on Figure 6.

For the workflow management skilled staff, laboratories of pharmacokinetics and pharmacodynamics, knowledge in the area of personalized medicine among the general practitioners and health professionals is needed.

There are stages of formation of such centers of personalized medicine:

1. The formation of a contract with FSBI Scientific Center of Assessment of Medical Products under MHSD of Russian Federation, I.M. Sechenov First Moscow State Medical University, P.A. Herzen Moscow Research Oncological Institute.

2. The information (in the form of methodical documents and lectures) about the drugs that are metabolized with the involvement of the isoenzymes of the cytochrome P-450 with the information about reasons that can enhance and weaken the isoenzymes activity should be given to the general practitioners of MPI.

3. The development of the working scheme between a general practitioner of the MPI and a coordinator (consultant of the center). At first, a general practitioner sends by e-mail case history and specific questions for the consultant of the center to answer, connected with pharmacotherapy (the choice of drug and dosage regimen). The consultant's work is compared with the duty in the hospital and should be paid in accordance with these principles.

### Personalized medicine is an innovative and science-intensive instrument for the improvement of the effectiveness and safety of pharmacotherapy of the socially significant diseases

The Europe key staffs of clinical pharmacology continually made an emphasis on timelines of this direction for the practical healthcare:

– The first president of the European Association for Clinical Pharmacology and Therapeutics (EACPT), emeritus professor of the Karolinska University (Stockholm, Sweden) and a member of the Nobel Committee for medicine, Folke Sjöqvist: ‘The personalized medicine is a preferred direction in Europe nowadays’.

– The ex-president of the European Association for Clinical Pharmacology and Therapeutics (EACPT) and the director of the Institute of Pharmacology (Kiel, Germany), professor Ingolf Cascorbi: ‘The personalized medicine is a rapidly developing discipline, aimed to the objective scientific basis for the studying the personal differences in drug reactions’.

## Competing interests

The authors declare that they have no competing interests.

## Authors’ contributions

ASZ, VGK, and DAS conceived of the study and participated in its design and coordination. All authors read and approved the final manuscript.

## Authors’ information

A current paper follows the recommendations of the EPMA White Paper.
